# Pathogen Spectrum and Antimicrobial Susceptibility Profiles in Culture-Proven Endophthalmitis at a Tertiary Referral Center in China: A Three-Decade Retrospective Study

**DOI:** 10.3390/antibiotics15070663

**Published:** 2026-07-06

**Authors:** Wenfei Zhang, Zhe Yang, Jingjia Zhang, Xinyu Zhao, Chenghui Peng, Yuelin Wang, Youxin Chen, Huan Chen

**Affiliations:** 1 Department of Ophthalmology, Peking Union Medical College Hospital, Chinese Academy of Medical Sciences and Peking Union Medical College, No. 1 Shuaifuyuan, Dongcheng District, Beijing 100730, China; zhangwenfei@pumch.cn (W.Z.); yangzhe@student.pumc.edu.cn (Z.Y.);; 2 Beijing Key Laboratory of Fundus Diseases Intelligent Diagnosis & Drug/Device Development and Translation, Beijing 100730, China; 3 Key Laboratory of Ocular Fundus Diseases, Chinese Academy of Medical Sciences, Beijing 100730, China; 4 Department of Clinical Laboratory, Peking Union Medical College Hospital, Chinese Academy of Medical Sciences, Beijing 100730, China; 5 Operating Room, Peking Union Medical College Hospital, Chinese Academy of Medical Sciences and Peking Union Medical College, Beijing 100730, China; 6 Department of Ophthalmology, Peking University Third Hospital, Beijing 100191, China

**Keywords:** endophthalmitis, antimicrobial susceptibility, prophylaxis

## Abstract

**Background/Objectives**: Endophthalmitis is a devastating, vision-threatening intraocular infectious disease. Given the scarcity of long-term cohort data in Asian populations, appropriate empirical antibiotic therapy is essential to prevent irreversible vision loss. The present study aimed to identify the culture-positive pathogen profiles and antimicrobial susceptibility in endophthalmitis. **Methods**: This retrospective study included 90 culture-positive isolates from patients with endophthalmitis treated between January 1990 and October 2020. The etiology, clinical presentation, and antibiotic susceptibility profiles of the isolates were analyzed. **Results**: Forty-three isolates were Gram-positive cocci, 23 were Gram-negative bacilli, and 24 were fungi. Postoperative infections were dominated by Gram-positive cocci (72.1%). Furthermore, 47.8% (11/23) of Gram-negative bacilli and 66.7% (16/24) of fungi were recovered from endogenous endophthalmitis isolates. In patients with endophthalmitis caused by Gram-negative bacilli, visual acuity was numerically inferior at both initial presentation and final follow-up visit (*p* = 0.051 and *p* = 0.018). All isolates of Gram-positive cocci exhibited full susceptibility to vancomycin and teicoplanin, while linezolid yielded a susceptibility rate of 96.9% (31/32). For Gram-negative bacilli, over 80% of isolates were susceptible to amikacin, aztreonam, and third-generation cephalosporins, whereas carbapenems and fluoroquinolones achieved susceptibility rates exceeding 90%. Among staphylococcal isolates, ciprofloxacin resistance was significantly higher in patients older than 55 years compared with those aged 55 years or younger (71.4% versus 12.5%; exact *p* = 0.013). **Conclusions**: Postoperative infection was the leading cause of Gram-positive cocci endophthalmitis. Antimicrobial susceptibility profiles validated intravitreal vancomycin combined with ceftazidime as a rational regimen for empirical antibacterial coverage. Elevated fluoroquinolone resistance among Gram-positive cocci, particularly in elderly patients, underscores the necessity of sustained regional antimicrobial resistance surveillance and periodic reassessment of conventional topical antibiotic prophylatic protocols.

## 1. Introduction

Endophthalmitis is a severe intraocular infection and remains an ophthalmic emergency because structural ocular damage and irreversible visual loss may occur rapidly. Since clinical findings may overlap among different causative organisms and culture results are not immediately available, initial management usually requires prompt intravitreal broad-spectrum antimicrobial therapy, commonly vancomycin for Gram-positive organisms plus ceftazidime for Gram-negative organisms, with vitrectomy considered according to disease severity and visual status [1].

The pathogen spectrum of endophthalmitis differs substantially across clinical settings. Acute postoperative endophthalmitis is most often caused by Gram-positive organisms, particularly coagulase-negative staphylococci [2]. A recent multicenter study also showed that Gram-positive bacteria predominated in culture-positive acute-onset postoperative endophthalmitis, with Staphylococcus epidermidis and other coagulase-negative staphylococci as leading isolates [3].

Regional and etiological differences further complicate empirical antimicrobial selection. A large cohort from southern China showed that pathogen distribution varied by clinical category. Gram-positive cocci were common in postoperative and post-traumatic endophthalmitis, whereas keratitis-associated endophthalmitis was mainly caused by filamentous fungi and Gram-negative bacilli [4]. Another study comparing children and adults also reported age-related differences in etiology and microbiological isolates, with Streptococcus species being more common in pediatric post-traumatic cases and coagulase-negative staphylococci predominating in adults [5]. These findings suggest that pathogen profiles cannot be generalized across regions, age groups, or clinical settings.

In parallel with pathogen variation, antimicrobial resistance has become an important concern in ocular infections. Although vancomycin generally remains active against most Gram-positive endophthalmitis isolates, susceptibility to fluoroquinolones, cephalosporins, aminoglycosides, and other commonly used agents may vary over time and across institutions. Recent culture-positive endophthalmitis data have also suggested age-related variation in both etiological distribution and antibiotic susceptibility, with increasing resistance observed in older patients [6].

Despite increasing reports from individual regions, long-term data integrating pathogen spectrum, clinical etiology, visual outcomes, and antimicrobial susceptibility remain limited in China. Longitudinal surveillance of ocular pathogens and their susceptibility profiles is important for validating existing empirical treatment protocols. Two previous studies from the same institutional database described the clinical characteristics and outcomes of the overall endophthalmitis cohort and the endogenous endophthalmitis subgroup, respectively [7,8]. The present study is a secondary, isolate-level analysis focused on pathogen-specific antibacterial susceptibility and fungal MIC profiles, which were not systematically reported previously. We aimed to describe pathogen-specific clinical patterns and antimicrobial susceptibility profiles, and assess their implications for empirical treatment and topical antibiotic prophylaxis.

## 2. Results

### 2.1. Clinical Characteristics

A total of 90 isolates with susceptibility data were included: 43 Gram-positive cocci, 23 Gram-negative bacilli, and 24 fungi. Clinical characteristics are summarized in Table 1. The etiologies of endophthalmitis varied significantly depending on the causative pathogens (*p* < 0.001). Post-surgery was the predominant cause for Gram-positive cocci (72.1%), while endogenous infections were the primary causes for Gram-negative bacilli and fungi (47.8% and 66.7%, respectively). The median initial logMAR best corrected visual acuity (BCVA) for Gram-positive cocci, Gram-negative bacilli, and fungi was 2.30 (2.30–2.70), 2.70 (2.30–2.85), and 2.30 (2.30–2.70), respectively (*p* = 0.051), while the final logMAR BCVA was 1.90 (0.70–2.70), 2.70 (2.30–3.00), and 2.50 (2.00–2.70), respectively (*p* = 0.018). In patients with endophthalmitis caused by Gram-negative bacilli, both initial and final BCVA were the worst. The initial treatment modalities included evisceration/enucleation, vitrectomy, and intravitreal injection (IVI) of antibiotics. For patients infected with Gram-negative bacilli, the proportion of those initially receiving evisceration/enucleation was higher compared to the other two groups (*p* = 0.032). After treatment, patients infected with Gram-positive cocci showed a significant improvement in visual acuity (*p* < 0.001). No significant within-group change was observed in the Gram-negative bacillary group (median, 2.70 vs. 2.70; *p* = 0.636) or the fungal group (median, 2.30 vs. 2.50; *p* = 0.241).

Detailed organism distributions are shown in Supplementary Tables S1–S3. Among the Gram-positive cocci, *Staphylococcus epidermidis* was the predominant species, accounting for 41.9% (18/43) of isolates. A total of 31 isolates (72.1%) were from post-operation cases, including 12 after cataract surgery, 8 after intravitreal injections, 6 after glaucoma surgery, 4 after vitrectomy, and 1 after combined cataract surgery and vitrectomy. The numbers of traumatic and endogenous endophthalmitis were 9 and 3, respectively. Among the Gram-negative bacilli, *Klebsiella pneumoniae* was the most frequent species, accounting for 39.1% (9/23), including six isolates associated with liver abscesses. *Candida albicans* was the most common fungal species, accounting for 45.8% (11/24) of fungal isolates. The single Gram-positive bacillary isolate was identified as *Enterocloster clostridioformis*. Because several organisms were represented by only one or two isolates, the broad pathogen categories in Table 1 were used only for descriptive comparisons of etiology and clinical outcomes, whereas antimicrobial susceptibility was reported at the species level or using taxonomically appropriate organism groups whenever possible.

### 2.2. Antibiotic Susceptibility of Gram-Positive Cocci

Overall susceptibility data for Gram-positive cocci were summarized in Table 2, and complete results were provided in Supplementary Table S4. All tested Gram-positive cocci were susceptible to imipenem (8/8), teicoplanin (29/29), vancomycin (41/41), rifampicin (24/24), and tigecycline (11/11), while 96.9% (31/32) of Gram-positive cocci were susceptible to linezolid. Susceptibility to gentamicin, oxacillin, ciprofloxacin, levofloxacin, and moxifloxacin was 57.1% (20/35), 50.0% (11/22), 42.9% (9/21), 57.1% (16/28), and 60.0% (3/5), respectively. Resistance was common to penicillin G (28/40, 70.0%), azithromycin (5/6, 83.3%). For erythromycin, 9/41 isolates (22.0%) were susceptible, 3/41 (7.3%) were intermediate, and 29/41 (70.7%) were resistant.

Species- and organism-group-level results showed that *Staphylococcus epidermidis* remained highly susceptible to cefmetazole (5/5), ceftriaxone (4/4), cefoperazone (5/5), and cefotaxime (4/5), with clindamycin susceptibility in 10/12 (83.3%). *Enterococcus faecalis* was uniformly susceptible to linezolid (4/4), ampicillin (4/4), and penicillin G (5/5) but resistant to gentamicin (4/4), erythromycin (4/4), and tetracycline (5/5). Viridans group *streptococci* were susceptible to linezolid in 4/4 tested isolates, to ceftriaxone, cefotaxime, and cefepime in 4/5 each, to chloramphenicol in 3/3, and to levofloxacin in 4/5. Results for *Streptococcus pneumoniae* and the single Group G β-hemolytic streptococcus are presented separately in Table 2.

**Figure 1:**
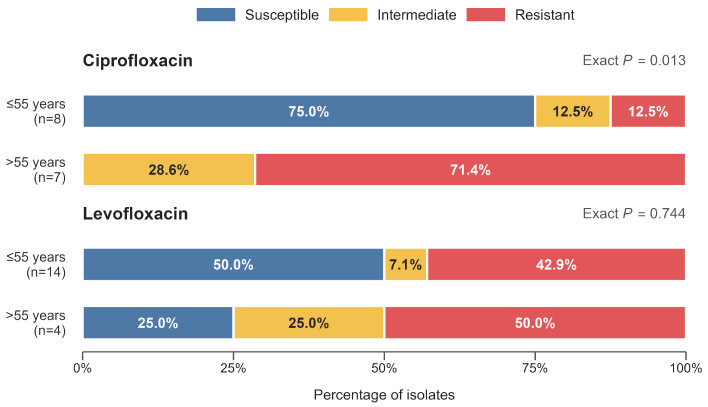
Age-stratified fluoroquinolone susceptibility among staphylococcal isolates. The stacked bars show the proportions of isolates classified as susceptible, intermediate, or resistant to ciprofloxacin and levofloxacin among patients aged ≤55 and >55 years.

Age-stratified fluoroquinolone susceptibility results were presented separately for *staphylococci, enterococci*, and *streptococci* in Table 3, and the distributions of ciprofloxacin and levofloxacin susceptibility among staphylococcal isolates were illustrated in Figure 1. Among the staphylococcal isolates, the distribution of ciprofloxacin susceptibility differed between patients aged ≤55 and >55 years, with susceptible isolates accounting for 75.0% (6/8) and 0% (0/7), respectively (*p* = 0.013). No significant age-related difference was observed for levofloxacin susceptibility (*p* = 0.744), while the number of isolates tested for moxifloxacin was insufficient for statistical comparison. All enterococcal isolates with available fluoroquinolone results were obtained from patients older than 55 years. Streptococcal results were reported descriptively because of the small and uneven numbers of isolates tested.

### 2.3. Antibiotic Susceptibility of Gram-Negative Bacilli

For 23 Gram-negative bacilli isolates, the susceptibility to amikacin and aztreonam was 83.3% (15/18) and 82.4% (14/17), respectively. For the third- and fourth-generation cephalosporins, the susceptibility was above 80% (ceftazidime 95.5%, ceftriaxone 90.0%, cefoperazone 88.9% and cefepime 100.0%). Among the 22 strains tested against ceftazidime, one strain of *Burkholderia cepacia* was found to be not susceptible to it, as well as to piperacillin-tazobactam and amikacin, while exhibiting susceptibility to imipenem. More than 90% of Gram-negative bacilli were susceptible to carbapenems (Ertapenem 11/11, Imipenem 18/20, Meropenem 16/16) and fluoroquinolones (Levofloxacin 14/15, Ciprofloxacin 20/21). The two imipenem-resistant strains were identified as *Morganella morganii* and *Pseudomonas fluorescens*, while both strains remained susceptible to ceftazidime (Table 4 and Table S5).

Nine *Klebsiella pneumoniae* isolates were susceptible to the following antibiotics: amikacin (9/9), aztreonam (9/9), cefoxitin (5/5), ceftazidime (9/9), cefoperazone (8/8), cefepime (9/9), carbapenems (9/9), piperacillin-sulbactam (9/9). Furthermore, 8/9 (88.9%) were susceptible to gentamicin, piperacillin, amoxicillin-clavulinic acid, ampicillin-sulbactam, ciprofloxacin and levofloxacin.

### 2.4. Antifungal Susceptibility of Fungal Isolates

Among the 24 fungal isolates, 14 (58.3%) were yeasts and 10 (41.7%) were molds. The yeasts comprised *Candida albicans* (*n* = 11), *Candida tropicalis* (*n* = 1), *Candida parapsilosis* (*n* = 1), and *Cryptococcus* sp. (*n* = 1). The molds comprised *Aspergillus fumigatus* (*n* = 3), *Aspergillus flavus* (*n* = 1), *Aspergillus niger* (*n* = 1), *Paecilomyces lilacinus* (*n* = 2), *Cladosporium* sp. (*n* = 1), *Fusarium* sp. (*n* = 1), and *Exophiala dermatitidis* (*n* = 1).

Fluconazole, voriconazole, itraconazole, and amphotericin B MIC results were available for 12, 22, 23, and 24 isolates, respectively. Flucytosine MIC results were available for 11 yeast isolates, and all were reported as ≤4 μg/mL. The corresponding median MICs for the remaining four antifungal agents were 1 μg/mL (range, 1–16), 0.06 μg/mL (range, 0.016–3), 0.23 μg/mL (range, 0.016 to >32), and 0.5 μg/mL (range, 0.032 to >32), respectively.

Among the 13 *Candida* isolates, 10 of 11 isolates with available fluconazole MICs had values ≤ 1 μg/mL, whereas one *C. albicans* isolate had an MIC of 4 μg/mL. All 11 isolates tested for 5-flucytosine had MICs ≤ 4 μg/mL. Voriconazole MICs ranged from 0.016 to 0.5 μg/mL, while itraconazole and amphotericin B MICs were ≤0.25 and ≤0.5 μg/mL, respectively. Among the molds, amphotericin B MICs were more variable. *Paecilomyces lilacinus* had low voriconazole MICs (0.023 and 0.032 μg/mL, respectively) but amphotericin B MICs > 32 μg/mL. The Cladosporium and Fusarium isolates also showed high itraconazole and amphotericin B MICs. Complete isolate-level results are provided in Supplementary Table S6.

## 3. Discussion

In our study, the common etiologies varied among the three types of pathogens, with post-surgery being the most common cause for Gram-positive cocci, while endogenous infection predominated for the other two groups. Visual acuity was worst in cases of endophthalmitis caused by Gram-negative bacilli, with no significant improvement observed post-treatment. Among the three types of pathogens, the most common were *Staphylococcus epidermidis*, *Klebsiella pneumoniae*, and *Candida albicans*, respectively. The susceptibility of Gram-positive cocci to imipenem, teicoplanin, vancomycin, rifampicin and tigecycline was 100%, followed by linezolid. About half of the Gram-positive cocci were susceptible to gentamicin, oxacillin and fluoroquinolones, and most of them were resistant to penicillin G and macrolides. Among staphylococcal isolates, the resistance to ciprofloxacin increased significantly in patients aged >55 years old. More than 80% of Gram-negative bacilli were susceptible to amikacin, aztreonam, third- and fourth-generation cephalosporins, and more than 90% were susceptible to carbapenems and fluoroquinolones. The high susceptibility rate and safety profile of vancomycin and ceftazidime supported their continued use as empirical antibiotics in the treatment of acute endophthalmitis in our hospital. Both Gram-positive cocci and Gram-negative bacilli were susceptible to imipenem, which has been reported to have a broad antibacterial spectrum, good penetration of the vitreous after systemic application and non-toxic to ocular structures. However, the application of intravitreal injection of imipenem was limited in clinical practice [9,10].

In Gram-positive cocci, coagulase-negative *Staphylococcus* was the most common isolated strain in post-operative and traumatic endophthalmitis [11]. In our study, *Staphylococcus epidermidis* was the most commonly encountered, reflecting the significant role of normal skin flora in endophthalmitis. Consistent with other studies, favorable visual outcomes could be achieved with prompt and aggressive treatment of the infection [12]. For endophthalmitis caused by Gram-negative bacilli, endogenous infection was the primary etiology, with *Klebsiella pneumoniae*-associated bacteremia and endophthalmitis being the most predominant. In East Asia, liver abscess has been confirmed as a significant contributor to extraocular infections, accounting for 19–57.5% of cases [13]. In our study, four cases initially undergoing evisceration/enucleation were infected with *Klebsiella pneumoniae.* Other studies have reported a similar trend, indicating poor visual outcomes associated with *Klebsiella pneumoniae*-related endogenous endophthalmitis. The primary factor is *K. pneumoniae*’s propensity to form expansive subretinal abscesses in the early stages, leading to retinal destruction and subsequent central vision loss. Furthermore, as this damage was concealed by an opaque vitreous, the extent of retinal necrosis was often underestimated [14]. Similarly, fungal endophthalmitis primarily originated from endogenous *Candida* infections. *Candida* is a constituent of the human microbiota, residing symbiotically on mucosal surfaces of the respiratory, gastrointestinal, and female reproductive systems. Reported risk factors included intravenous drug use, prolonged hospitalization, indwelling venous catheters, immunosuppressive conditions and others [15].

The overall susceptibility of Gram-negative bacilli to fluoroquinolones was higher than that of Gram-positive cocci [16]. While different articles may utilize varying age thresholds, it is widely acknowledged that the susceptibility of Gram-positive bacteria to fluoroquinolones significantly decreases with age. The increased utilization of topical perioperative antibiotic prophylaxis, particularly fluoroquinolones, is associated with a concomitant surge in antibiotic resistance [17], and older patients are more exposed to a wide variety of antibiotics [6]. The fourth-generation fluoroquinolones gatifloxacin and moxifloxacin could achieve therapeutic levels in aqueous humor for many organisms. However, topical application of these agents failed to achieve vitreous MIC90 levels. This called into question the recommended use of these antibiotics in routine prophylaxis [18]. Linezolid is a synthetic oxazolidinone antibiotic to which 96.9% of the Gram-positive cocci in our study were susceptible. Linezolid has been shown to have good intraocular penetration when administered orally and to be nontoxic to rabbit eyes after an intraocular injection of 0.40 mg [19]. Similarly, in the study by Gentile RC et al., the susceptibility of Gram-positive isolates to linezolid was 99.3%. Cases of endophthalmitis caused by vancomycin-resistant strains have been reported, with *Staphylococcus* and *Enterococcus* being the predominant pathogens, in addition to other species such as *Streptococcus*, *Bacillus,* and *Leuconostoc.* These infections were often associated with poor visual outcomes. The broad coverage spectrum of linezolid against Gram-positive bacteria suggests its potential as an alternative to vancomycin in the future, particularly as vancomycin resistance becomes increasingly prevalent. Khera M et al. reported seven cases of vancomycin-resistant Gram-positive organisms, among which four strains were susceptible to amikacin [20]. Amikacin was primarily used to treat Gram-negative bacteria. Nevertheless, its use was justified in cases of infections caused by Gram-positive microorganisms that exhibited susceptibility exclusively to amikacin [21]. In addition, alternative antibiotics mentioned in the literature for endophthalmitis caused by vancomycin-resistant Gram-positive bacteria included quinupristin/dalfopristin, daptomycin, tigecycline, and other agents to which the microorganism demonstrated susceptible based on susceptibility testing [22,23,24].

Gram-negative bacilli were highly susceptible to ceftazidime, amikacin, and fluoroquinolones. Aminoglycoside antibiotics such as amikacin and gentamicin can be effective against Gram-negative endophthalmitis, but their retinal toxicity has also been fully demonstrated [25]. In our study, the susceptibility of Gram-negative bacilli to third-generation fluoroquinolones was above 90%. Intravitreal injection of fluoroquinolones has been proposed as an empirical intravitreal antibiotic [26]. It may therefore be possible in the future to combine these agents with antibiotics that are active against Gram-positive organisms.

The fungal isolates showed substantial species-level heterogeneity. Most *Candida* isolates had relatively low MICs to the tested azoles and amphotericin B, whereas several filamentous fungi showed markedly higher MICs to itraconazole or amphotericin B. In particular, *Paecilomyces lilacinus* had low voriconazole MICs but amphotericin B MICs > 32 μg/mL. These findings support species-level identification and antifungal susceptibility testing in fungal endophthalmitis. Amphotericin B has traditionally been the first-line antifungal agent for fungal endophthalmitis. The usual intravitreous injection dose ranged from 5 to 10 μg (peak vitreous concentration, 1.25 to 2.50 μg/mL), which has been shown to be safe in both phakic and aphakic eyes, as well as in vitrectomized eyes [27]. Fluconazole is an older triazole, usually administered orally (200 to 400 mg/day). A single oral dose (400 mg) of fluconazole can achieve an average peak plasma concentration of 6.72 μg/mL (4.12 to 8.08 μg/mL), and an assumed vitreous concentration of 4.70 μg/mL (2.88–5.66 μg/mL) [28]. Voriconazole is a triazole that became available in 2002, and intravitreal injection of voriconazole (100 μg) is considered as the standard, with a peak intravitreal concentration of 25 μg/mL and a half-life of 2.5 h. According to the Infectious Diseases Society of America (IDSA), intravitreal injection of amphotericin B (5–10 µg/0.1 mL sterile water) or voriconazole (100 µg/0.1 mL sterile water or saline) was recommended for *Candida* endophthalmitis. At the same time, fluconazole/voriconazole (susceptible) and liposomal AmB (fluconazole/voriconazole-resistant) were used as systemic drugs [29]. Nevertheless, direct comparison between in vitro MICs and achievable ocular drug concentrations should be made cautiously. The present fungal findings were based on small numbers of isolates, testing methods varied during the study period, and established clinical breakpoints were unavailable for several uncommon ocular fungal pathogens.

This study has several limitations. First, generalized antimicrobial susceptibility breakpoints were used. Because ocular antibiotic concentrations after local ocular administration, particularly intravitreal injection, could be higher than those after systemic treatment, resistance may be overestimated. In addition, CLSI interpretive breakpoints were revised several times during the 30-year study period. Complete retrospective standardization using a single contemporary breakpoint system was not feasible because original quantitative susceptibility measurements were not uniformly available for all historical isolates. Second, the analysis was restricted to culture-positive isolates because culture-negative cases provided neither an identifiable organism nor antimicrobial susceptibility data. The present findings should not be generalized to culture-negative endophthalmitis. Third, several species and organism–antimicrobial combinations were represented by small numbers, and susceptibility testing panels were not uniform across isolates, resulting in variable denominators and limiting robust species-specific or temporal analyses. Finally, fungal susceptibility testing was performed using several reference and commercial methods during the study period. The differences in MIC measurements may therefore have contributed to the observed variability. Accordingly, the above findings should be regarded as descriptive rather than as standardized temporal resistance estimates.

## 4. Materials and Methods

This retrospective study reviewed patients with endophthalmitis admitted to Peking Union Medical College Hospital between January 1990 and October 2020. The study adhered to the Declaration of Helsinki and was approved by the Ethics Committee of Peking Union Medical College Hospital (approval number K3924).

Vitreous and aqueous humor samples were processed in the hospital microbiology laboratory. Bacterial cultures were performed on 5% sheep blood agar, chocolate agar, and MacConkey agar. Organism identification and antimicrobial susceptibility interpretations were abstracted from the original clinical microbiology reports. Patients with clinically diagnosed but culture-negative endophthalmitis were excluded because no identifiable microbial isolate or antimicrobial susceptibility result was available for the pathogen-specific analyses. The source population partially overlapped with the cohorts reported previously [7,8]. However, the microbial isolate, rather than the patient or affected eye, was the principal unit of analysis in the present study. Bacterial susceptibility testing used the Kirby–Bauer method. Susceptible, intermediate, and resistant categories were abstracted from the original clinical microbiology reports and reflected the CLSI or predecessor interpretive criteria in effect at the time of testing. For each organism–antimicrobial combination, the denominator included only isolates with an explicit susceptible, intermediate, or resistant interpretation. Because original inhibition zone diameters and quantitative MIC values were not uniformly available for historical isolates, the results could not be retrospectively reinterpreted using a single contemporary breakpoint system. Fungal susceptibility testing methods varied according to the organism and study period. Stock solutions of fluconazole, 5-flucytosine, voriconazole, itraconazole, and amphotericin B were prepared in dimethyl sulfoxide (DMSO; MedChemExpress, Monmouth Junction, NJ, USA). Clinical and Laboratory Standards Institute (CLSI)-recommended methods for antifungal susceptibility test of filamentous fungi were consistently employed by our clinical microbiology laboratory. With the introduction of commercialized methods, Etest and ATB methods were also adopted. For susceptibility testing of yeast, the disk diffusion method was previously employed, limited to azole drugs. Subsequently, the CLSI-recommended broth microdilution method was utilized, along with commercial Etest and YeastOne plates. The minimum inhibitory concentration (MIC) was determined by comparison with drug-free growth control wells: azole with a 50% reduction in yeast turbidity and a 100% reduction in mold turbidity; amphotericin B with a 100% reduction in turbidity for all isolates, and 5-flucytosine with a 50% reduction in yeast turbidity. Interpretation of microbial growth followed the CLSI. The guidelines used to interpret the susceptibility of bacteria, yeast, and filamentous fungi were CLSI M100, CLSI M27, and CLSI M38, respectively [30,31,32]. Patients with clinically diagnosed but culture-negative endophthalmitis were excluded because no viable microbial isolate or antimicrobial susceptibility result was available for the pathogen-specific analyses.

Statistical analysis was performed by SPSS 26.0 (SPSS Inc., Chicago, IL, USA). Continuous variables were assessed for distributional characteristics. Normally distributed variables are presented as mean ± standard deviation and were compared using parametric tests, whereas non-normally distributed variables are presented as median and interquartile range and were compared using non-parametric tests, as appropriate. Paired continuous data were analyzed using the Wilcoxon signed-rank test when the assumptions of parametric testing were not met. Categorical variables were compared using the chi-square test or Fisher’s exact test, and continuous variables using independent *t*-tests or non-parametric tests as appropriate. A *p* value < 0.05 was considered statistically significant.

## 5. Conclusions

In this three-decade, single-center cohort of culture-positive endophthalmitis, pathogen distribution differed according to clinical etiology. Postoperative infection predominated among Gram-positive coccal isolates, whereas endogenous infection accounted for a substantial proportion of Gram-negative bacillary and fungal isolates. The observed bacterial susceptibility profiles support the continued use of intravitreal vancomycin plus ceftazidime for empirical antibacterial coverage at our center. Given the widespread perioperative use of topical fluoroquinolones, the observed fluoroquinolone resistance among Gram-positive cocci warrants continued local surveillance and careful selection of prophylactic antibiotics according to institutional susceptibility patterns. Larger multicenter studies are needed to confirm these findings and inform empirical treatment and prophylactic strategies.

## Figures and Tables

**Table 1:** Clinical Information of Endophthalmitis Caused by Different Pathogens.

	Gram-Positive Cocci (*N* = 43)	Gram-Negative Bacilli (*N* = 23)	Fungi (*N* = 24)	*p*
Age (range)	52.5 ± 18.9 (2–79)	52.4 ± 17.5 (6–75)	48.2 ± 12.6 (21–64)	0.381
Male (%)	30 (69.8)	14 (60.9)	10 (41.7)	0.080
**Causes**				
Post-surgery (%)	31 (72.1%)	6 (26.1%)	5 (20.8%)	
Trauma (%)	9 (20.9%)	6 (26.1%)	3 (12.5%)	
Endogenous infection (%)	3 (7.0%)	11 (47.8%)	16 (66.7%)	**<0.001**
Initial logMAR BCVA, median (IQR)	2.30 (2.30–2.70)	2.70 (2.30–2.85)	2.30 (2.30–2.70)	**0.051**
**Initial treatment option**				
Evisceration/enucleation (%)	1 (2.3)	5 (21.7%)	0	
Vitrectomy/IVI	42 (97.7)	18 (78.3%)	24 (100.0)	**0.032**
Final logMAR BCVA, median (IQR)	1.90 (0.70–2.70)	2.70 (2.30–3.00)	2.50 (2.00–2.70)	**0.018**

BCVA = best corrected visual acuity; IVI = intravitreal injection of antibiotics.

**Table 2:** Antimicrobial susceptibility of Gram-positive cocci to major clinically relevant agents.

	*Staphylococcus* *epidermidis* (*n* = 18) No. (%)	*Staphylococcus* *aureus* (*n* = 4)	Other Coagulase-Negative *Staphylococci* * (*n* = 8)	*Enterococcus* *faecalis* (*n* = 5)	Viridans Group *streptococci* (*n* = 5) #	*Streptococcus pneumoniae* (*n* = 2)	Group G β-Hemolytic *streptococcus* (*n* = 1)
**Aminoglycosides**							
Gentamicin	10/18 (55.6)	2/4	8/8	0/4	/	/	R
**Oxazolidinones**							
Linezolid	12/13 (92.3)	4/4	6/6	4/4	4/4	1/1	/
**β-lactams**							
Ampicillin	0/4	/	0/1	4/4	/	/	S
Oxacillin	4/11	3/3	3/7	/	/	/	S
Piperacillin	2/3	/	1/1	1/1	/	/	/
Penicillin G	2/18 (11.1)	4/4	0/8	5/5	3/3	1/1	S
Cefazolin	5/7 (71.4)	/	4/7	/	/	/	S
Ceftriaxone	4/4	/	1/3	/	4/5	/	S
**Fluoroquinolones**							
Ciprofloxacin	5/9 (55.6)	0/2	1/4	3/5	/	/	I
Levofloxacin	4/9 (44.4)	2/2	2/7	2/3	4/5	1/1	S
Moxifloxacin	0/2	1/1	/	/	1/1	1/1	/
**Glycopeptides**							
Teicoplanin	12/12	4/4	7/7	5/5	/	1/1	/
Vancomycin	17/17	4/4	8/8	5/5	5/5	1/1	S
**Macrolides**							
Erythromycin	6/17 (35.3)	1/4	2/8	0/4	0/5	0/2	R
Trimethoprim-Sulfamethoxazole	9/13 (69.2)	2/4	3/7	/	/	1/2	S

Data are presented as the number of susceptible isolates divided by the number of isolates tested, with percentages shown in parentheses. S, susceptible; I, intermediate; R, resistant. A slash indicates that no susceptibility result was available. Complete agent- and species-specific susceptibility results are provided in Supplementary Table S4. * Other coagulase-negative *staphylococci* included five isolates not identified to the species level and one isolate each of *Staphylococcus haemolyticus*, *Staphylococcus lugdunensis*, and *Staphylococcus hominis.* # Viridans group *streptococci* included two isolates of *Streptococcus parasanguinis*, one isolate of *Streptococcus oralis*, and two isolates not identified beyond the viridans group level.

**Table 3:** Age-stratified fluoroquinolone susceptibility among Gram-Positive Cocci.

Bacterial Group	Fluoroquinolone	≤55 Years	S, n (%)	I, n (%)	R, n (%)	>55 Years	S, n (%)	I, n (%)	R, n (%)	*p* Value
*Staphylococci* (*n* = 30)	Ciprofloxacin	8	6 (75.0)	1 (12.5)	1 (12.5)	7	0 (0.0)	2 (28.6)	5 (71.4)	0.013
	Levofloxacin	14	7 (50.0)	1 (7.1)	6 (42.9)	4	1 (25.0)	1 (25.0)	2 (50.0)	0.744
	Moxifloxacin	2	1 (50.0)	0 (0.0)	1 (50.0)	1	0 (0.0)	1 (100.0)	0 (0.0)	/
*Enterococci* (*n* = 5)	Ciprofloxacin	0	—	—	—	5	3 (60.0)	0 (0.0)	2 (40.0)	/
	Levofloxacin	0	—	—	—	3	2 (66.7)	0 (0.0)	1 (33.3)	/
*Streptococci* (*n* = 8)	Ciprofloxacin	0	—	—	—	1	0 (0.0)	1 (100.0)	0 (0.0)	/
	Levofloxacin	3	3 (100.0)	0 (0.0)	0 (0.0)	4	3 (75.0)	0 (0.0)	1 (25.0)	/
	Moxifloxacin	2	2 (100.0)	0 (0.0)	0 (0.0)	0	—	—	—	/

S, susceptible; I, intermediate; R, resistant. Percentages were calculated using the number of isolates tested for the corresponding fluoroquinolone within each bacterial and age group as the denominator. A dash indicates that no isolate in the corresponding group was tested. *p* values compare the distributions of susceptible, intermediate, and resistant isolates between the two age groups.

**Table 4:** Antimicrobial susceptibility of Gram-negative bacilli to major clinically relevant agents.

	*Klebsiella pneumoniae* (*n* = 9) No. (%)	*Pseudomonas aeruginosa* (*n* = 3)	*Escherichia coli* (*n* = 2)	*Burkholderia cepacia* (*n* = 2)	*Pseudomonas stutzeri* (*n* = 1)	*Plesiomonas shigelloides* (*n* = 1)	*Klebsiella oxytoca* (*n* = 1)	*Morganella morganii* (*n* = 1)	*Pseudomonas fluorescens* (*n* = 1)	*Acinetobacter lwoffii* (*n* = 1)	*Proteus mirabilis* (*n* = 1)
**Aminoglycosides**											
Amikacin	9/9	2/2	1/1	0/1	/	R	S	S	R	/	S
Gentamicin	8/9 (88.9)	2/2	1/2	0/1	I	I	/	R	R	S	R
**β-lactams**											
Aztreonam	9/9	2/2	1/1	0/1	/	/	S	R	/	I	S
Ceftazidime	9/9	3/3	2/2	0/1	S	S	S	S	S	S	S
Ceftriaxone	7/8 (87.5)	1/2	2/2	1/1	S	S	S	S	S	S	S
Cefoperazone	8/8	2/2	2/2	/	I	S	/	S	R	S	S
Cefotaxime	7/8 (87.5)	0/2	1/1	1/1	S	S	S	/	/	S	S
Cefepime	9/9	2/2	1/1	1/1	/	S	S	S	/	S	S
Imipenem	9/9	2/2	2/2	1/1	/	S	S	R	R	S	S
Meropenem	9/9	2/2	1/1	1/1	/	/	S	S	/	/	S
Piperacillin-Sulbactam	9/9	2/2	1/1	/	/	S	S	S	/	S	S
Ticarcillin-Clavulanic Acid	4/5 (80.0)	2/2	1/1	0/1	R	S	S	/	R	S	S
**Fluoroquinolones**											
Ciprofloxacin	8/9 (88.9)	2/2	2/2	1/1	S	S	S	S	S	S	S
Levofloxacin	8/9 (88.9)	2/2	1/1	/	/	/	S	S	/	/	S
Moxifloxacin	/	/	/	/	/	/	/	/	/	/	/
Sulfamethoxazole	5/8 (62.5%)	/	1/1	2/2	/	S	/	R	/	S	R
Tetracyclines											

Data are presented as the number of susceptible isolates divided by the number of isolates tested. For species represented by a single isolate, susceptibility results are expressed as S, I, or R. S = Sensitive; I = Intermediate; R = Resistant. A slash indicates that no susceptibility result was available. Complete agent- and species-specific susceptibility results are provided in Supplementary Table S5.

## Data Availability

The data presented in this study are available on request from the corresponding author due to privacy and ethical restrictions.

## Supplementary Materials

The following supporting information can be downloaded at: https://www.mdpi.com/article/10.3390/antibiotics15070663/s1, Table S1: Distributions of Gram-positive Cocci under Different Etiologies; Table S2: Distributions of Gram-negative Bacilli under Different Etiologies; Table S3: Distributions of Fungi under Different Etiologies; Table S4: Antibiotic Susceptibility of Gram-Positive Cocci; Table S5: Antibiotic Susceptibility of Gram-Negative Bacilli; Table S6: Minimum Inhibitory Concentrations of Fungal Cultures.
