# Supplementary material for: Pathogen Spectrum and Antimicrobial Susceptibility Profiles in Culture-Proven Endophthalmitis at a Tertiary Referral Center in China: A Three-Decade Retrospective Study
Source: Antibiotics (Basel). 2026 Jul 6;15(7):663. doi: 10.3390/antibiotics15070663 (PMC13403405; doi:10.3390/antibiotics15070663)
Supplement: Supplementary file 1 [file antibiotics-15-00663-s001.zip › antibiotics-4361192-supplementary.pdf]

Supplementary Table S1. Distributions of Gram-positive Cocci under Different Etiologies.

Causes		Number (%)
**Post-cataract surgery**		12 (27.9)
	Enterococcus faecalis	4 (9.3)
	Staphylococcus epidermidis	3 (7.0)
	Staphylococcus aureus	1 (2.3)
	Other coagulase-negative staphylococci	1 (2.3)
	Streptococcus parasanguinis	1 (2.3)
	Streptococcus pneumoniae	1 (2.3)
	Group G β-hemolytic streptococcus	1 (2.3)
**Post-intravitreal injection**		8 (18.6)
	Staphylococcus epidermidis	4 (9.3)
	Staphylococcus aureus	1 (2.3)
	Staphylococcus hominis	1 (2.3)
	Other coagulase-negative staphylococci	1 (2.3)
	Viridans group streptococcus	1 (2.3)
**Post-glaucoma surgery**		6 (14.0)
	Enterococcus faecalis	1 (2.3)
	Staphylococcus epidermidis	3 (7.0)
	Staphylococcus haemolyticus 1	1 (2.3)
	Viridans group streptococcus	1 (2.3)
**Post-vitrectomy**		4 (9.3)
	Staphylococcus epidermidis	3 (7.0)
	Other coagulase-negative staphylococci	1 (2.3)
**Post-combined cataract surgery and vitrectomy**		1 (2.3)
	Staphylococcus epidermidis	1 (2.3)
**Trauma**		9 (20.9)
	Staphylococcus epidermidis	4 (9.3)
	Staphylococcus lugdunensis	1 (2.3)
	Other coagulase-negative staphylococci	2 (4.6)
	Streptococcus parasanguinis	1 (2.3)
	Streptococcus pneumoniae	1 (2.3)
**Endogenous infection**		3 (7.0)
	Staphylococcus aureus	2 (4.6)
	Streptococcus oralis	1 (2.3)

Supplementary Table S2. Distributions of Gram-negative Bacilli under Different Etiologies.

Causes	Number (%)
**Post-cataract surgery**	1 (4.3)
Burkholderia Cepacia	1 (4.3)
**Post-glaucoma surgery**	2 (8.7)
Pseudomonas aeruginosa	1 (4.3)
Proteus mirabilis	1 (4.3)
**Post-vitrectomy**	2 (8.7)
Burkholderia Cepacia	1 (4.3)
Morganella morganii	1 (4.3)
**Post-corneal ulcer**	1 (4.3)
Pseudomonas aeruginosa	1 (4.3)
**Trauma**	6 (26.1)
Pseudomonas stutzeri	1 (4.3)
Escherichia coli	1 (4.3)
Plesiomonas shigelloides	1 (4.3)
Klebsiella oxytoca	1 (4.3)
Pseudomonas fluorescens	1 (4.3)
Acinetobacter lwoffii	1 (4.3)
**Endogenous infection**	11 (47.8)
Klebsiella pneumoniae	9 (39.1)
Escherichia coli	1 (4.3)
Pseudomonas aeruginosa	1 (4.3)

Supplementary Table S3. Distributions of Fungi under Different Etiologies.

Causes	Number (%)
**Post-cataract surgery**	2 (8.3)
Fusarium	1 (4.2)
Exophiala dermatitidis	1 (4.2)
**Post-intravitreal injection**	1 (4.2)
Candida tropicalis	1 (4.2)
**Post-scleral buckling**	2 (8.3)
Aspergillus fumigatus	1 (4.2)
Aspergillus *flavus*	1 (4.2)
**Trauma**	3 (12.5)
Cladosporium	1 (4.2)
Aspergillus niger	1 (4.2)
Candida parapsilosis	1 (4.2)
**Endogenous infection**	16 (66.7)
Candida albicans	11 (45.8)
Paecilomyces lilacinus	2 (8.3)
Aspergillus fumigatus	2 (8.3)
Cryptococcus	1 (4.2)

Supplementary Table S4. Antibiotic Susceptibility of Gram-Positive Cocci.

	Staphylococcus epidermidis (n=18) No. (%)	*Staphylococcus aureus* (n=4)	Other Coagulase-negative staphylococci* (n=8)	*Enterococcus faecalis* (n=5)	Viridans group streptococci (n = 5)#	*Streptococcus pneumoniae* (n=2)	Group G β-hemolytic streptococcus (n=1)
**Aminoglycosides**							
Gentamicin	10/18 (55.6)	2/4	8/8	0/4	/	/	R
**Oxazolidinones**							
Linezolid	12/13 (92.3)	4/4	6/6	4/4	4/4	1/1	/
**β-lactams**							
Oxacillin	4/11	3/3	3/7	/	/	/	S
Penicillin G	2/18 (11.1)	4/4	0/8	5/5	3/3	1/1	S
Cefoxitin	4/11 (36.4)	3/3	0/5	/	/	/	/
Cefotaxime	4/5	/	3/3	/	4/5	/	S
Imipenem	4/4	/	1/1	2/2	/	/	S
Amoxicillin-Clavulanic Acid	4/5	2/2	3/4	2/2	/	/	S
Ampicillin-sulbactam	2/4	/	1/4	3/3	/	/	/
Chloramphenicol	/	/	2/3	1/4	3/3	1/2	S
Clindamycin	10/12 (83.3)	3/4	5/7	/	1/5	0/2	R
**Fluoroquinolones**							
Ciprofloxacin	5/9 (55.6)	0/2	1/4	3/5	/	/	I
Levofloxacin	4/9 (44.4)	2/2	2/7	2/3	4/5	1/1	S

Moxifloxacin	0/2	1/1	/	/	1/1	1/1	/
**Glycopeptides**							
Teicoplanin	12/12	4/4	7/7	5/5	/	1/1	/
Vancomycin	17/17	4/4	8/8	5/5	5/5	1/1	S
**Macrolides**							
Erythromycin	6/17 (35.3)	1/4	2/8	0/4	0/5	0/2	R
**Rifampicin**	13/13	4/4	5/5	2/2	/	/	/
Trimethoprim-Sulfamethoxazole	9/13 (69.2)	2/4	3/7	/	/	1/2	S
**Tetracyclines**							
Tetracycline	7/9 (77.8)	2/3	2/3	0/5	1/2	0/2	R
Tigecycline	7/7	1/1	1/1	/	1/1	1/1	/

Data are presented as the number of susceptible isolates divided by the number of isolates tested, with percentages shown in parentheses. S, susceptible; I, intermediate; R, resistant. A slash indicates that no susceptibility result was available.

*Other coagulase-negative staphylococci included five isolates not identified to the species level and one isolate each of Staphylococcus haemolyticus, Staphylococcus lugdunensis, and Staphylococcus hominis.

#Viridans group streptococci included two isolates of Streptococcus parasanguinis, one isolate of Streptococcus oralis, and two isolates not identified beyond the viridans group level.

Supplementary Table S5. Antibiotic Susceptibility of Gram-Negative Bacilli.

	Klebsiella pneumoniae (n=9) No. (%)	Pseudomonas aeruginosa (n=3)	Escherichia coli (n=2)	Burkholderia Cepacia (n=2)	Pseudomonas stutzeri (n=1)	Plesiomonas shigelloides (n=1)	Klebsiella oxytoca (n=1)	Morganella morganii (n=1)	Pseudomonas fluorescens (n=1)	Acinetobacter lwoffii (n=1)	Proteus mirabilis (n=1)	Gram-negative bacilli No. (%)
**Aminoglycosides**												
Amikacin	9/9	2/2	1/1	0/1	/	R	S	S	R	/	S	15/18 (83.3)
Gentamicin	8/9 (88.9)	2/2	1/2	0/1	I	I	/	R	R	S	R	12/20 (60.0)
**β-lactams**												
Aztreonam	9/9	2/2	1/1	0/1	/	/	S	R	/	I	S	14/17 (82.4)
Piperacillin	8/9 (88.9)	2/2	/	0/1	/	R	S	/	R	S	S	13/17 (76.5)
Cefazolin	/	/	1/1	0/1	R	S	/	/	S	R	R	3/7 (42.9)
Cefmetazole	3/4 (75.0)	/	1/1	/	S	/	/	/	S	/	/	6/7 (85.7)
Cefoxitin	5/5	/	1/1	1/1	/	S	/	I	/	S	R	9/11 (81.8)
Cefuroxime	6/9 (66.7)	/	2/2	/	R	S	S	/	S	S	R	12/17 (70.6)
Ceftazidime	9/9	3/3	2/2	0/1	S	S	S	S	S	S	S	21/22 (95.5)
Ceftriaxone	7/8 (87.5)	1/2	2/2	1/1	S	S	S	S	S	S	S	18/20 (90.0)
Cefoperazone	8/8	2/2	2/2	/	I	S	/	S	R	S	S	16/18 (88.9)
Cefotaxime	7/8 (87.5)	0/2	1/1	1/1	S	S	S	/	/	S	S	14/17 (82.4)
Cefepime	9/9	2/2	1/1	1/1	/	S	S	S	/	S	S	18/18 (100.0)
Ertapenem	9/9	/	1/1	/	/	/	/	S	/	/	/	11/11 (100.0)
Imipenem	9/9	2/2	2/2	1/1	/	S	S	R	R	S	S	18/20 (90.0)

Meropenem	9/9	2/2	1/1	1/1	/	/	S	S	/	/	S	16/16 (100.0)
Amoxicillin-Clavulanic Acid	8/9 (88.9)	/	1/1	/	/	S	/	/	/	/	R	10/12 (83.3)
Ampicillin-sulbactam	8/9 (88.9)	/	1/1	/	S	/	/	/	S	/	R	11/13 (84.6)
Piperacillin-Sulbactam	9/9	2/2	1/1	/	/	S	S	S	/	S	S	17/18 (94.4)
Ticarcillin-Clavulanic Acid	4/5 (80.0)	2/2	1/1	0/1	R	S	S	/	R	S	S	11/15 (73.3)
**Fluoroquinolones**												
Ciprofloxacin	8/9 (88.9)	2/2	2/2	1/1	S	S	S	S	S	S	S	20/21 (95.2)
Levofloxacin	8/9 (88.9)	2/2	1/1	/	/	/	S	S	/	/	S	14/15 (93.3)
Moxifloxacin	/	/	/	/	/	/	/	/	/	/	/	/
Macrolides	/	/	/	/	/	/	/	/	/	/	/	/
Sulfamethoxazole	5/8 (62.5%)	/	1/1	2/2	/	S	/	R	/	S	R	10/15 (66.7)
**Tetracyclines**												
Minocycline	6/8 (75.0)	/	1/1	1/1	/	/	/	/	/	/	/	8/10 (80.0)
Tigecycline	5/7 (71.4)	/	1/1	/	/	/	/	/	/	/	/	6/8 (75.0)

Data are presented as the number of susceptible isolates divided by the number of isolates teste. For species represented by a single isolate, susceptibility results are expressed as S, I, or R. S=Sensitive; I=Intermediate; R=Resistant. A slash indicates that no susceptibility result was available.

Supplementary Table S6. Minimum Inhibitory Concentrations of Fungal Cultures.

Case	Year of Diagnosis	Organism	Fluconazole	5-flucytosine	Voriconazole	Itraconazole	Amphotericin B
1	2007	*Candida albicans*	/	/	0.016μg/mL	0.016μg/mL	0.032μg/mL
2	2008	*Candida albicans*	/	/	/	0.23μg/mL	0.23μg/mL
3	2009	*Candida albicans*	≤1μg/mL	≤4μg/mL	≤0.06μg/mL	≤0.125μg/mL	≤0.5μg/mL
4	2012	*Candida albicans*	≤1μg/mL	≤4μg/mL	≤0.06μg/mL	≤0.125μg/mL	≤0.5μg/mL
5	2014	*Candida albicans*	≤1μg/mL	≤4μg/mL	≤0.06μg/mL	≤0.125μg/mL	≤0.5μg/mL
6	2015	*Candida albicans*	≤1μg/mL	≤4μg/mL	≤0.06μg/mL	≤0.125μg/mL	≤0.5μg/mL
7	2018	*Candida albicans*	≤1μg/mL	≤4μg/mL	≤0.06μg/mL	≤0.125μg/mL	≤0.5μg/mL
8	2018	*Candida albicans*	≤1μg/mL	≤4μg/mL	≤0.06μg/mL	≤0.125μg/mL	≤0.5μg/mL
9	2019	*Candida albicans*	≤1μg/mL	≤4μg/mL	≤0.06μg/mL	≤0.125μg/mL	≤0.5μg/mL
10	2019	*Candida albicans*	4μg/mL	≤4μg/mL	0.5μg/mL	0.25μg/mL	≤0.5μg/mL
11	2020	*Candida albicans*	≤1μg/mL	≤4μg/mL	≤0.06μg/mL	≤0.125μg/mL	≤0.5μg/mL
12	2011	*Candida parapsilosis*	≤1μg/mL	≤4μg/mL	≤0.06μg/mL	≤0.125μg/mL	≤0.5μg/mL
13	2010	*Candida tropicalis*	≤1μg/mL	≤4μg/mL	≤0.06μg/mL	≤0.125μg/mL	≤0.5μg/mL
14	2011	*Aspergillus fumigatus*	/	/	0.094μg/mL	0.5μg/mL	0.047μg/mL

15	2015	*Aspergillus fumigatus*	/	/	/	1.5μg/mL	0.125μg/mL
16	2017	*Aspergillus fumigatus*	/	/	0.047μg/mL	0.38μg/mL	16μg/mL
17	2016	Aspergillus *flavus*	/	/	0.1μg/mL	≤0.5μg/mL	1μg/mL
18	2006	*Aspergillus niger*	/	/	0.125μg/mL	0.5μg/mL	0.094μg/mL
19	2012	*Paecilomyces lilacinus*	/	/	0.023μg/mL	/	>32μg/mL
20	2019	*Paecilomyces lilacinus*	/	/	0.032μg/mL	1.5μg/mL	>32μg/mL
21	2006	*Cladosporium*	/	/	3μg/mL	32μg/mL	>32μg/mL
22	2009	*Fusarium*	/	/	1μg/mL	>32μg/mL	8μg/mL
23	2012	*Cryptococcus*	16μg/mL	/	0.75μg/mL	0.75μg/mL	0.75μg/mL
24	2019	*Exophiala dermatitidis*	/	/	0.06μg/mL	0.64μg/mL	0.5μg/mL
